# Case of Dislocation of the Ulna Backward, Etc.

**Published:** 1874-03

**Authors:** Norman Bridge

**Affiliations:** Chicago


ARTICLE IV.—*A Case of Dislocation of the Ulna backward, without Displacement of the Radius.* Reported by NORMAN BRIDGE, M.D., Chicago.

J. S., a German of medium height and slight build, and twenty-five years old, was thrown from his wagon July 27th, 1873, and received an injury of his left elbow.

He was seen by the writer within twenty minutes after the accident. There was an apparent projection backward, of the olecranon; the arm was held at right angles; the hand was in the supine position, and bent laterally toward the ulnar side, as in the position assumed on dressing with the pistol splint. There was no abrasion externally, or apparent contusion.

On examination, the ulna was found dislocated backward, the radius not being displaced. No other abnormality could be made out.

Under the influence of ether the dislocation was reduced, very little force being required to replace the bones, and a distinct sound being produced as the joint surfaces struck together. A light dressing was applied for protection, and the arm placed in a sling.

There was for a few days considerable swelling and heat, for which water dressings were applied. In a week the man began to use the arm, and in a month very little effect of the injury remained. The contour of the joint was perfect on recovery.

In view of the exceeding infrequency of this accident, and the difficulty of its production without fracture of the coronoid process of the ulna, it may be questioned whether in this case such a complication did not exist.

I am only sure there was no crepitation on manipulating the elbow after the displacement was reduced; nor was there any other indication that to me positively suggested a fracture.

That there was extensive rupture of the ligaments, if no fracture existed, may be presumed from the ease with which reduction was accomplished. At the time the case was examined no considerable swelling had taken place, and the position of the head of the radius, the condylis of the humerus and the olecranon, were distinctly demonstrated.

